# A new pairwise kernel for biological network inference with support vector machines

**DOI:** 10.1186/1471-2105-8-S10-S8

**Published:** 2007-12-21

**Authors:** Jean-Philippe Vert, Jian Qiu, William S Noble

**Affiliations:** 1 Centre for Computational Biology, Ecole des Mines de Paris, 35 rue Saint-Honoré, 77300 Fontainebleau, France; 2 Department of Genome Sciences, University of Washington, 1705 NE Pacific Street, Seattle, WA 98195, USA; 3 Department of Computer Science and Engineering, University of Washington, 1705 NE Pacific Street, Seattle, WA 98195, USA

## Abstract

**Background:**

Much recent work in bioinformatics has focused on the inference of various types of biological networks, representing gene regulation, metabolic processes, protein-protein interactions, etc. A common setting involves inferring network edges in a supervised fashion from a set of high-confidence edges, possibly characterized by multiple, heterogeneous data sets (protein sequence, gene expression, etc.).

**Results:**

Here, we distinguish between two modes of inference in this setting: direct inference based upon similarities between nodes joined by an edge, and indirect inference based upon similarities between one pair of nodes and another pair of nodes. We propose a supervised approach for the direct case by translating it into a distance metric learning problem. A relaxation of the resulting convex optimization problem leads to the support vector machine (SVM) algorithm with a particular kernel for pairs, which we call the *metric learning pairwise kernel*. This new kernel for pairs can easily be used by most SVM implementations to solve problems of supervised classification and inference of pairwise relationships from heterogeneous data. We demonstrate, using several real biological networks and genomic datasets, that this approach often improves upon the state-of-the-art SVM for indirect inference with another pairwise kernel, and that the combination of both kernels always improves upon each individual kernel.

**Conclusion:**

The metric learning pairwise kernel is a new formulation to infer pairwise relationships with SVM, which provides state-of-the-art results for the inference of several biological networks from heterogeneous genomic data.

## Background

Increasingly, molecular and systems biology is concerned with describing various types of subcellular networks. These include protein-protein interaction networks, metabolic networks, gene regulatory and signaling pathways, and genetic interaction networks. While some of these networks can be partly deciphered by high-throughput experimental methods, fully constructing any such network requires lengthy biochemical validation. Therefore, the automatic prediction of edges from other available data, such as protein sequences, global network topology or gene expression profiles, is of importance, either to speed up the elucidation of important pathways or to complement high-throughput methods that are subject to high levels of noise [1].

Edges in a network can be inferred from relevant data in at least two complementary ways. For concreteness, consider a network of protein-protein interactions derived from some noisy, high-throughput technology. Our confidence in the correctness of a particular edge *A *- *B *in this network increases if we observe, for example, that the two proteins *A *and *B *localize to the same cellular compartment or share similar evolutionary patterns [2-4]. Generally, in this type of *direct inference*, two genes or proteins are predicted to interact if they bear some direct similarity *to each other *in the available data.

An alternative mode of inference, which we call *indirect inference*, relies upon similarities between pairs of genes or proteins. In the example above, our confidence in *A *- *B *increases if we find some other, high-confidence edge *C *- *D *such that the pair {*A*, *B*} resembles {*C*, *D*} in some meaningful fashion. Note that in this model, the two connected proteins *A *and *B *might not be similar to one another. For example, if the goal is to detect edges in a regulatory network by using time series expression data, one would expect the time series of the regulated protein to be delayed in time compared to that of the regulatory protein. Therefore, in this case, the learning phase would involve learning this feature from other pairs of regulatory/regulated proteins. The most common application of the indirect inference approach in the case of protein-protein interaction involves comparing the amino acid sequences of *A *and *B *versus *C *and *D *(e.g., [5-8]).

Indirect inference amounts to a straightforward application of the machine learning paradigm to the problem of edge inference: each edge is an example, and the task is to learn to discriminate between "true" and "false" edges. Not surprisingly, therefore, several machine learning algorithms have been applied to predict network edges from properties of protein pairs. For example, in the context of machine learning with support vector machines (SVM) and kernel methods, Ben-Hur and Noble [8] describe how to map an embedding of individual proteins onto an embedding of pairs of proteins. The mapping defines two pairs of proteins as similar to each other when each protein in a pair is similar to one corresponding protein in the other pair. In practice, the mapping is defined by deriving a kernel function on pairs of proteins from a kernel function on individual proteins, obtained by a tensorization of the initial feature space. We therefore call this pairwise kernel the *tensor product pairwise kernel *(TPPK, see Methods section).

Less attention has been paid to the use of machine learning approaches in the direct inference paradigm. Two exceptions are the works of Yamanishi *et al*. [9] and Vert *et al*. [10], who derive supervised machine learning algorithms to optimize the measure of similarity that underlies the direct approach by learning from examples of interacting and non-interacting pairs. Yamanishi *et al*. employ kernel canonical correlation analysis to embed the proteins into a feature space where distances are expected to correlate with the presence or absence of interactions between protein pairs. Vert *et al*. highlight the similarity of this approach with the problem of distance metric learning [11], while proposing an algorithm for that purpose.

Both of these direct inference approaches, however, suffer from two important drawbacks. First, they are based on the optimization of a proxy function that is slightly different from the objective of the embedding, namely, finding a distance metric such that interacting/non-interacting pairs fall above/below some threshold. Second, the methods of [9] and [10] are applicable only when the known part of the network used for training is the set of all edges among a subset of proteins in the network. In other words, in order to apply these methods, we must have a complete set of high-confidence edges for one set of proteins, from which we can infer edges in the rest of the network by assuming that edges not observed among the proteins in the training set are really absent. This setting is often unrealistic. In practice, our training data will generally consist of known positive and negative edges distributed throughout the target network. For example, in the case of protein-protein interactions, one typically derives positive examples of interactions from experimental assays, while negative examples can be sampled randomly among non-interacting pairs or generated from pairs of proteins known to be present in different cellular localization or expressed under different conditions; the methods of [9] and [10] can not be used in this setting.

In this paper we propose a convex formulation for supervised learning in the direct inference paradigm that overcomes both of the limitations mentioned above. This formulation stems from a particular formulation of the distance metric learning problem [10,11]. We show that a slight relaxation of this formulation bears surprising similarities with the supervised approach of [8], in the sense that it amounts to defining a kernel between pairs of proteins from a kernel between individual proteins. We therefore call our method the *metric learning pairwise kernel *(MLPK). An important property of this formulation as an SVM is the possibility to learn from several data types simultaneously by combining kernels, which is of particular importance in various bioinformatics applications [12,13].

Several authors have proposed algorithms for distance metric learning with kernels related to our method. Tsang and Kwok [14] propose a quadratic program (QP) formulation of the problem, while Weinberger *et al*. [15] propose a semidefinite programming formulation in the context of distance metric learning for *k*-nearest-neighbour classifiers. In both cases, however, a specific algorithm must be implemented. To the contrary, the formulation we propose builds upon the well-known SVM algorithm. Any practitioner of SVM can therefore easily use it with most public SVM implementations, at the price of using a specific kernel. A second advantage of our SVM formulation is that it can be easily combined with other SVM formulation, such as the TPPK approach, by forming linear combinations of different kernels.

We validate the MLPK approach on the task of reconstructing two yeast networks: the network of metabolic pathways and the co-complex network. In each case, the network is inferred from a variety of genomic and proteomic data, including protein amino acid sequences, gene expression levels over a large set of experiments, and protein subcellular localization. We show that the MLPK approach nearly always provides better prediction performance than the state-of-the-art TPPK approach, and that the combination of the MLPK and TPPK together almost always leads to the best results.

## Results and discussion

In this section we present a comparison of the previously described TPPK kernel and the new MLPK kernel for the reconstruction of two biological networks: the metabolic network and the co-complex protein network. For each network, we cast the problem of network reconstruction as a binary classification problem, where the presence or absence of edges must be inferred from various types of data relevant to the problem. Because the network contains relatively few edges compared to the total number of possible pairs, we created a balanced dataset by keeping all known edges as positive examples and randomly sampling an equal number of absent edges as negative examples. We compare the utilities of the TPPK and MLPK kernels in this context by assessing the performance of an SVM for edge prediction in a five-fold cross-validation experiment repeated three times (3 × 5 cv) with different random folds. At each fold, the regularization parameter *C *of the SVM is chosen among 18 values evenly log-spaced on the interval [10^-4^, 50] by minimizing the classification error estimated by five-fold cross-validation within the training set only. We also assess the performance of the pairwise kernel obtained by summing the TPPK and MLPK kernels, which we call *MLPK *+ *TTPK *below. The MLPK + TPPK kernel is a simple way to combine the information contained in the MLPK and TTPK kernels. We also test two approaches to integrate the various genomic and proteomic data for edge prediction. First we construct an integrated kernel over genes, obtained by adding together all kernels defined by the various data, and deduce a TPPK, MLPK or MLPK + TTPK pairwise kernel from this integrated kernel. This is a simple approach to data integration that has proved useful in previous work [12,16]. Alternatively, we consider the pairwise kernels deduced from each individual genomic data, and add them together to form an integrated pairwise kernel.

As a baseline method for direct inference, for each kernel between genes we also assess the performance of a direct method that ranks the candidate edges by increasing distance between the two gene involved, where the distance between two genes is derived from the kernel value by the equation:

$$d_{K}(x,y)=\sqrt{K(x,x)+K(y,y)-2K(x,y)}.$$

### Metabolic network

Most biochemical reactions in living organisms are catalyzed by particular proteins called enzymes, and occur sequentially to form metabolic pathways. For example, the degradation of glucose into pyruvate (called glycolysis) involves a sequence of ten chemical reactions catalyzed by ten enzymes. The metabolic gene network is defined as an undirected graph with enzymes as vertices and with edges connecting pairs of enzymes that can catalyze successive chemical reactions. The reconstruction of metabolic pathways for various organisms is of critical importance, e.g., to find new ways to synthesize chemical compounds of interest. This problem motivated earlier work on supervised graph inference [9,10]. Focusing on the budding yeast *S. cerevisiae*, we collected the metabolic network and genomic data used in [9]. The network was extracted from the KEGG database and contains 769 vertices and 3702 undirected edges.

In order to infer the network, various independent data about the proteins can be used. In this experiment, we use four relevant sources of data provided by [9]: (1) a set of 157 gene expression measurements obtained from DNA microarrays; (2) the phylogenetic profiles of the genes, represented as 145-bit vectors indicating the presence or absence of each gene in 145 fully sequenced genomes; (3) the protein's localization in the cell determined experimentally [17], represented as 23-bit vectors corresponding to 23 cellular compartments, and (4) yeast two-hybrid protein-protein interaction data [1], represented as a network. For the first three data sets, a Gaussian RBF kernel was used to represent the data as a kernel matrix. For the yeast two-hybrid network, we use a diffusion kernel [18]. All data were downloaded from 

Table 1 shows the performance of each pairwise kernel, as well as that of the baseline direct approach, for the different data sets. The MLPK is never worse than the TPPK kernel, and both methods are always much better than the baseline direct method for edge inference. The two kernels have similar performance on the sum kernel; MLPK is slightly better than TPPK on the expression, localization and phylogenetic profile kernels, and much better on the yeast two-hybrid dataset (76.6% vs. 59.2% in accuracy). Finally we observe that the integrated kernel MLPK + TPPK is always at least as good as the best of MLPK or TPPK alone, confirming that MLPK and TTPK are complementary to one another.

**Table 1:** Performance on reconstruction of the yeast metabolic networks.

	MLPK		TPPK		MLPK + TPPK		Direct
Data	Accuracy	AUC	Accuracy	AUC	Accuracy	AUC	AUC
Expression	77.9 ± 1.2	84.8 ± 1.2	77.4 ± 0.9	84.1 ± 0.4	78.2 ± 0.9	84.9 ± 1.3	51.9 ± 1.6
Localization	63.8 ± 2.2	67.5 ± 3.0	62.4 ± 1.0	65.6 ± 0.8	64.4 ± 0.9	66.3 ± 1.0	55.1 ± 1.4
Phylogenetic profile	79.5 ± 0.9	84.3 ± 0.9	77.7 ± 1.6	83.6 ± 1.7	80.7 ± 0.8	85.4 ± 1.1	60.7 ± 1.4
Yeast two-hybrid	75.9 ± 1.2	82.5 ± 1.4	59.4 ± 1.0	65.4 ± 1.7	76.7 ± 0.8	83.0 ± 0.4	51.6 ± 1.4
Sum	83.9 ± 0.7	91.6 ± 0.5	84.0 ± 0.7	91.2 ± 0.4	83.9 ± 0.9	91.5 ± 0.6	60.6 ± 1.3
Pairwise sum	81.4 ± 0.5	89.0 ± 0.4	80.7 ± 1.1	88.6 ± 0.6	81.6 ± 0.7	89.2 ± 0.8	-

The table lists, for each type of data, the accuracy and area under the ROC curve obtained by each pairwise kernel. Values in the tables are means and standard errors in a 3 × 5 cv experiment. TPPK is the tensor product pairwise kernel, and MLPK is the metric learning pairwise kernel. The column MLPK + TTPK shows the results when an SVM is trained with the sum of the MLPK and TPPK pairwise kernels. The row *Sum *shows the results when the kernel between the genes is the sum of the expression, localization, phylogenetic profile and yeast two-hybrid kernels. The line *Pairwise sum *shows the results obtained with the SVM when the pairwise kernel used is the sum of pairwise kernels derived from the expression, localization, phylogenetic profile and yeast two-hybrid kernels, respectively. The *Direct *column shows the result of the direct method, where gene pairs are ranked according to their distance as defined by each kernel to predict edges.

Interestingly, we note that although connected pairs, i.e., pairs of enzymes acting successively in a pathway, are expected to have similar expression, phylogenetic profiles and localization (explaining the good performance of the MLPK on these datasets), the indirect approach implemented by the TPPK also gives good results for these data. This result implies that for these data, interacting pairs in the training set are often similar not only to each other but also to other interacting pairs in the training set. This observation is not surprising because, for example, if two proteins in the test set are co-localized in a particular organelle, then it is likely that interacting pairs of proteins co-localized in the same organelle are also present in the training set.

In the case of yeast two-hybrid data, on the other hand, the kernel between single proteins is defined as a diffusion kernel over the yeast two-hybrid graph. One can speculate that, in that case, similarity between pairs can be easily assessed and used by the MLPK to predict edges, but similarity between pairs as defined by the TPPK kernel is less likely to be observed. In a sense, the dimensionality of the feature space of the diffusion kernels is much larger than that defined by the other kernels, and a protein is only close to its neighbors in the yeast two-hybrid graph.

Regarding the integration of heterogeneous data sets, the pairwise kernels deduced from the sum of the individual kernels performs slightly better than the sum of the pairwise kernels deduced from individual kernels, which performs itself always better than the best of the pairwise kernels deduced from individual kernels. This confirms that the simple addition of kernels is a simple and powerful means to learn from heterogeneous data, and shows that in the case of pairwise kernels it seems better to first integrate heterogeneous data at the level of individual genes, before converting this integrated kernel into a pairwise kernel.

### Protein complex network

Many proteins carry out their biological functions by acting together in multi-protein structures known as complexes. Understanding protein function therefore requires identification of these complexes. In the co-complex network, nodes are proteins, and an edge between proteins *A *and *B *exists if *A *and *B *are members of the same protein complex. Some high-throughput experimental methods, such as tandem affinity purification followed by mass spectrometry, explicitly identify these co-complex relationships, albeit in a noisy fashion. Also, computational methods exist for inferring the co-complex network from individual data types or from multiple data types simultaneously [19,20]. We derived the co-complex data set based on an intersection of the manually curated MIPS complex catalogue [21] and the BIND complex data set [22]. The co-complex network contains 3280 edges connecting 797 proteins. In addition, our data set contains 3081 proteins with no co-complex relationships.

For this evaluation, we again use four different data sets that we consider relevant to the co-complex network. The first data set is the same localization data that we used above [17]. The second is derived from a chip-based version of the chromatin immunoprecipitation assay (so-called "ChIP-chip" data) [23]. This assay provides evidence that a transcription factor binds to the upstream region of a given gene and is likely to regulate the expression of the given gene. Our data set contains data for 113 transcription factors, and so yields a vector of length 113 for each protein. The final two data sets are derived from the amino acid sequences of the yeast proteins. For the first, we compared each yeast protein to every model in the Pfam database of protein domain HMMs (pfam.wustl.edu) and recorded the E-value of the match. This comparison yields a vector of length 8183 for each protein. Finally, in a similar fashion, we compared each yeast protein to each protein in the Swiss-Prot database version 40 (ca.expasy.org/sprot) using PSI-BLAST [24], yielding vectors of length 101,602. Each of the four data sets is represented using a scalar product kernel.

We used the same experimental procedure to compare the quality of edge predictors for the co-complex network using MLPK, TPPK and their combination MLPK + TPPK. The results, shown in Table 2, again show the value of the MLPK approach. Using either performance metric (accuracy or ROC area), the MLPK approach performs better than the TPPK approach on three out of four data sets. Both methods strongly outperform the direct approach on all datasets.

**Table 2:** Performance on reconstruction of the yeast co-complex networks.

	MLPK		TPPK		MLPK + TPPK		Direct
Data	Accuracy	AUC	Accuracy	AUC	Accuracy	AUC	AUC
Localization	76.2 ± 1.0	76.9 ± 2.0	79.5 ± 1.8	82.9 ± 1.7	80.6 ± 0.7	83.0 ± 1.2	73.9 ± 1.4
Chip-chip	82.2 ± 1.1	89.7 ± 0.8	63.8 ± 1.2	68.0 ± 1.1	84.4 ± 1.2	90.8 ± 1.2	58.4 ± 1.5
Pfam	92.1 ± 0.9	98.0 ± 0.5	86.1 ± 1.0	91.8 ± 0.9	93.8 ± 0.3	98.5 ± 0.1	67.3 ± 1.2
PSI-BLAST	89.0 ± 0.9	97.0 ± 0.1	88.3 ± 1.0	93.5 ± 0.9	93.1 ± 0.6	97.9 ± 0.2	67.8 ± 1.2
Sum	93.6 ± 0.3	98.7 ± 0.2	94.1 ± 0.6	98.0 ± 0.3	95.8 ± 0.3	99.1 ± 0.3	79.9 ± 0.8
Pairwise sum	93.3 ± 0.8	98.2 ± 0.4	90.5 ± 0.9	96.3 ± 0.7	95.2 ± 0.3	98.9 ± 0.2	-

The table lists, with the notation conventions explained in Figure 1, the results of the different methods on the reconstruction of the yeast co-complex networks.

Most striking is the improvement for the ChIP-chip data set (accuracy from 63.8% to 82.2%). This result is expected, because we know that proteins in the same complex must act in concert. As such, they are typically regulated by a common set of transcription factors.

In contrast, the MLPK approach does not perform better than TPPK on the localization data set. This is, at first, suprising because two proteins must co-localize in order to participate in a common complex. This problem is thus an example of the direct inference case for which the MLPK is designed. However, the localization data is somewhat complex because (1) only approximately 70% of yeast proteins are assigned any localization at all, and (2) many proteins are assigned to multiple locations. As a result, among 3280 positive edges in the training set, only 1852 (56%) of those protein pairs share exactly the same localization. Furthermore, 550 (16.8%) of the 3280 negative edges used in training connect proteins with the same localization, primarily "Unknown." These factors make direct inference using this data set difficult. The indirect method, by contrast, is apparently able to identify useful relationships, corresponding to specific localizations, that are enriched among the positive pairs relative to the negative pairs.

The fact that the MLPK and TPPK capture complementary information is further demonstrated by the good performance of the combined MLPK + TPPK approach, which is always better than both TPPK and MLPK alone on all datasets. Finally, the relevance of heterogeneous data integration by kernel summation is again demonstrated by the excellent results obtained in this case, with a slight advantage to the construction of a pairwise kernel over the integrated kernel for genes. The combination of MLPK + TPPK over the integrated kernel results in the best performance.

## Conclusion

We showed that a particular formulation of metric distance learning for graph inference can be formulated as a convex optimization problem and can be applied to any data set endowed with a positive definite kernel. A relaxation of this problem leads to the SVM algorithm with the new MLPK kernel (5) between pairs. Experiments on two biological networks confirm the value of this approach for the reconstruction of biological network from heterogeneous genomic and proteomic data.

The MLPK kernel is derived from a new formulation for distance metric learning. Contrary to other formulations [14,15] the resulting algorithm is a classical SVM with a particular kernel. This formulation can therefore benefit from the popularity of SVM in the computational biology community coupled with the availability of numerous public implementations of SVM, to solve various problems of gene or protein network inference, or more generally pairwise relationships inference.

This formulation, however, is obtained at the price of relaxing a positive definiteness constraint for the sake of computational efficiency. While the experimental results validate the approach for practical gene network inference, the relaxed formulation can not be considered as a distance metric learning algorithm anymore, because the final metric matrix may have negative eigenvalues. This discrepancy between the motivation of our approach (formulating graph inference as distance metric learning) and the final algorithm might complicate the interpretation of the results obtained, and will be subject to further investigations in the future.

Beyond the direct and indirect approaches to graph inference mentioned in the introduction, there exist many alternative ways to infer networks, such as estimating conditional independence between vertices with Bayesian networks [25]. An interesting property of methods based on supervised learning, such as the SVM with the TPPK and MLPK kernels, is the limited hypothesis made on the nature of the edges; the only hypothesis made is that there is information related to the presence or absence of edges in the data, and we let the learning algorithm model this information. The good accuracy obtained on two completely different networks (metabolic and co-complex) supports the general utility of this approach.

An interesting and important avenue for future research is the extension of these approaches to inference of directed graphs, e.g., regulatory networks. Although the TPPK and MLPK approaches are not adapted as such to this problem, variants involving for example kernels between ordered pairs could be studied.

## Methods

In this section we first explain how SVM can be used for graph inference, present the TPPK and MLPK kernels and provide some intuitive analysis of their differences. We then provide a detailed derivation of the MLPK kernel in the context of distance metric learning. After explaining the link between graph inference and distance metric learning, we first propose a new algorithm for distance metric learning when the genomic data are represented by vectors. We then generalize this algorithm to the case where the data are not necessarily finite-dimensional vectors, but more generally when a positive definite kernel is defined over the vertices. Finally, we introduce a relaxation of the resulting optimization problem, and we show that the problem is then equivalent to an SVM for a particular pairwise kernel, which we explicitly identify as the MLPK.

### SVM and positive definite kernels

Our method for graph inference is based on the SVM algorithm, a widely-used algorithm for supervised binary classification [26,27]. Given a set of points *x*_1_,...,*x*_*n *_with binary labels *y*_1_,...,*y*_*n *_∈ {-1, 1}, SVM estimate a function:

$$f(x)=\sum_{i=1}^{n}\alpha_{i}K(x_{i},x)+b,$$

to predict the label of any new point *x *by the sign of *f*(*x*). The function *K *in (1) is the so-called *kernel*, which must be a symmetric and positive definite function (i.e., for any integer *p *and any set of points *u*_1_,...,*u*_*p *_the square *p *× *p *matrix *K*_*i*,*j *_= *K*(*u*_*i*_, *u*_*j*_) must be symmetric and positive semidefinite). The weights *α*_*i *_(*i *= 1,...,*n*) and offset *b *in (1) are obtained by solving the following quadratic program:

$$\underset{\alpha ,b,\zeta }{\min}\sum_{i,j=1}^{n}\alpha_{i}\alpha_{j}K(x_{i},x_{j})+C\sum_{i=1}^{n}\zeta_{i},$$

under the constraints

$$\begin{array}{l} \begin{array}{cc} \zeta_{i}\geq 0, & i=1,...,n \end{array},\\ \begin{array}{cc} \zeta_{i}\geq 1-y_{i}\left(\sum_{j=1}^{n}\alpha_{j}K(x_{j},x_{i})+b\right), & i=1,...,n. \end{array} \end{array}$$

An interesting property of SVM is the complete modularity between the choice of the kernel *K*, on the one hand, and the algorithm. In other words the same SVM implementation can be used to process different data and solve different problems by simply modifying the data and the kernel used.

### Pairwise kernels for graph inference

We formulate the problem of supervised graph inference as follows: given a set of known interacting and non-interacting pairs of genes, build a classification function to predict for all pairs not used in the training phase whether they interact or not. In order to formalize this problem let us assume that a gene is represented by a point *x*, and that a kernel *K *between genes has been chosen. This kernel can for example be derived from genomic data, such as a microarray expression profile. We consider a set of *n *genes *x*_1_,...,*x*_*n*_, and a training set T = ℐ ∪ N of interacting (ℐ) and non-interacting (N) pairs; our objective is to learn a function to predict which pairs outside the training set interact or not.

By labeling +1 interacting pairs and -1 non-interacting pairs, this problem is a classical binary supervised classification problem, which can be solved with a SVM as soon as a kernel is defined. The difficulty is that the patterns to be classified are *pairs *of genes, while we assume that only a kernel between *individual *genes is available.

Ben-Hur and Noble proposed in [8] a general formula to create a kernel between pairs or patterns from a kernel between individual patterns:

*K*_*TPPK*_((*x*_1_, *x*_2_), (*x*_3_, *x*_4_)) = *K*(*x*_1_, *x*_3_)*K*(*x*_2_, *x*_4_) + *K*(*x*_1_, *x*_4_)*K*(*x*_2_, *x*_3_).

The rationale behind this tensor product pairwise kernel (TPPK) is that the comparison between a pair (*x*_1_, *x*_2_) and another pair (*x*_3_, *x*_4_) is done through the comparison of *x*_1 _with *x*_3 _and *x*_2 _with *x*_4 _(using the kernel between individual genes), on the one hand, and the comparions of *x*_1 _with *x*_4 _and *x*_2 _with *x*_3_, on the other hand.

In this paper we propose another pairwise kernel as follows:

*K*_*MLPK*_((*x*_1_, *x*_2_), (*x*_3_, *x*_4_)) = (*K*(*x*_1_, *x*_3_) - *K*(*x*_1_, *x*_4_) - *K*(*x*_2_, *x*_3_) + *K*(*x*_2_, *x*_4_))^2^.

This metric learning pairwise kernel (MLPK) is justified in detail in the following subsections and its link with the problem of distance metric learning highlighted. Although the formula of the MLPK (5) might seem less intuitive than the TPPK (4), some simple algebra can help highlight their difference. Indeed, any positive definite kernel can be written as an inner product after embedding the points to some Hilbert space [28]:

*K*(*x*, *x'*) = Φ(*x*)^⊤^Φ(*x'*),

where Φ is the mapping from the space of pattern to the feature Hilbert space. Consequently the MLPK can be rewritten as follows by plugging (6) into (5):

*K*_*MLPK*_((*x*_1_, *x*_2_), (*x*_3_, *x*_4_)) = [(Φ(*x*_1_) - Φ(*x*_2_))^⊤^(Φ(*x*_3_) - Φ(*x*_4_))]^2^.

This equation suggests that, up to the square exponent, the MLPK is an inner product between pairs after mapping a pair (*x*_1_, *x*_2_) to the vector Φ(*x*_1_) - Φ(*x*_2_). Hence a major difference between the TPPK and MLPK is that the former involves comparison between individual genes of the first pair and individual genes of the second pair, while the later compares pairs through the differences between their elements (in the feature space). In particular two pairs might be very similar with respect to the MLPK kernel even if the patterns of the first pair are very different from the patterns of the second pair, resulting in a large dissimilarity with respect to the TPPK kernel.

The rest of this section is devoted to a more rigorous derivation of the MLPK kernel, in particular to show its relationship to distance metric learning

### Distance metric learning

Following [10], we note that a possible approach to solve the problem of graph inference is to learn a distance metric *d *between genes with the property that pairs of nearby genes with respect to *d *are connected by an edge, while pairs of genes far from each other are not. If such a metric is available, then the prediction of an edge between a candidate pair of genes simply amounts to computing their distance to each other and predicting an edge if the distance is below a threshold.

More formally, let us first assume that genes are represented by finite-dimensional vectors and investigate distance metrics obtained by linear transformations of the input space. Such metrics are indexed by symmetric positive semidefinite matrices *M *as follows:

*d*_*M*_(*x*, *x'*) = (*x *- *x'*)^⊤^*M*(*x *- *x'*).

Our goal is to learn a distance metric which separates interacting from non-interacting pairs, while controlling over-fitting to the training set. Following the spirit of the SVM algorithm, we enforce an arbitrary margin of 2 between the distances of interacting and non-interacting variables up to slack variables, and control the Frobenius norm of *M *by considering the following problem:

$$\underset{\gamma ,M,\zeta }{\min}{\left\|M\right\|}_{Fro}^{2}+C\sum_{(i,j)\in T}\zeta_{ij},$$

under the constraints:

$$\begin{array}{c} \begin{array}{cc} \zeta_{ij}\geq 0, & (i,j)\in T, \end{array}\\ \begin{array}{cc} d_{M}(x_{i},x_{j})\leq \gamma -1+\zeta_{ij}, & (i,j)\in ℐ, \end{array}\\ \begin{array}{cc} d_{M}(x_{i},x_{j})\geq \gamma +1-\zeta_{ij}, & (i,j)\in N, \end{array}\\ M≽0. \end{array}$$

In order to solve this problem we first prove the following extension to the representer theorem [29]:

#### Theorem 1

The solution of (8–9) can be expanded as:

$$M=\sum_{(i,j)\in T}\alpha_{ij}(x_{i}-x_{j}){\left(x_{i}-x_{j}\right)}^{⊤},$$

*with α_ij _*∈ ℝ *for *(*i*, *j*) ∈ T.

#### Proof

For any pair (*i*, *j*), let us denote *u*_*ij *_= *x*_*i *_- *x*_*j*_, and let *D*_*ij *_be the *p *× *p *matrix *D*_*ij *_= (*x*_*i *_- *x*_*j*_)(*x*_*i *_- *x*_*j*_)^⊤ ^= *u*_*ij*_$u_{ij}^{⊤}$. Then we can rewrite

*d*_*M*_(*x*_*i*_, *x*_*j*_) = ⟨*M*, *D*_*ij*_⟩_*Fro*_,

where ⟨*A*, *B*⟩_*Fro *_= *Trace*(*A*^⊤^*B*) is the Frobenius inner product. Introducing the hinge loss function *L*(*y*, *y'*) = max(1 - *yy'*, 0) for *y*, *y' *∈ ℝ, and the indicator variables:

$$y_{ij}=\{\begin{array}{ll} 1 & \text{if }(i,j)\in \text{N},\\ -1 & \text{if }(i,j)\in \text{I}, \end{array}$$

we can eliminate the slack variables and rewrite the problem (8–9) as:

$$\underset{M≽0,\gamma \in \mathbb{R}}{\min}{\left\|M\right\|}_{Fro}^{2}+C\sum_{(i,j)\in T}L({\langle M,D_{ij}\rangle }_{Fro}-\gamma ,y_{ij}).$$

This shows that the optimization problem is in fact equivalent, up to the positive semidefinitiveness constraint, to an SVM in the linear space of symmetric matrices endowed with the Frobenius inner product. Each edge example is then mapped to the matrix *D*_*ij*_. In particular, if the constraint on *M *was not present, then Theorem 1 would be exactly the representer theorem. Here we need to show that it still holds with the constraint *M *≽ 0. For this purpose let *M *≽ 0 and *γ *∈ ℝ be the solution of (8–9). *M *can be uniquely decomposed as *M *= *M*_*S *_+ *M*_⊥_, where *M*_*S *_is in the linear span of (*D*_*ij*_, (*i*, *j*) ∈ T) and ⟨*M*_⊥_, *D*_*ij*_⟩_*Fro *_= 0 for (*i*, *j*) ∈ T. By the Pythagorean theorem we have ${\left\|M\right\|}_{Fro}^{2}={\left\|M_{S}\right\|}_{Fro}^{2}+{\left\|M_{⊥}\right\|}_{Fro}^{2}$, so if *M*_⊥ _≠ 0 the functional minimized in (10) is strictly smaller at (*M*_*S*_, *γ*) than at (*M*, *γ*); this would be a contradiction if *M*_*S *_≽ 0. Therefore, to prove the theorem it suffices to show *M*_*S *_≽ 0. Let *v *∈ ℝ^*p *^be any vector. We can decompose that vector uniquely as *v *= *v*_*S *_+ *v*_⊥_, where *v*_*S *_is in the linear span of the *u*_*ij*_, (*i*, *j*) ∈ T and $v_{⊥}^{⊤}u_{ij}=0$ for (*i*, *j*) ∈ T. We then have *M*_*S*_*v*_⊥ _= 0 and *M*_⊥_*v*_*S *_= 0, and therefore

$$v^{⊤}M_{S}v=v_{S}^{⊤}M_{S}v_{S}=v_{S}^{⊤}M_{S}v_{S}+v_{S}^{⊤}M_{⊥}v_{S}=v_{S}^{⊤}Mv_{S}\geq 0,$$

where we used the fact that *M *≽ 0 in the last inequality. This is true for any *v *∈ ℝ^*p*^, which shows that *M*_*S*_≽ 0, concluding the proof.   ■

By plugging the result of Theorem 1 into (8–9) we see that this problem is equivalent to that of finding *α*_*ij*_, (*i*, *j*) ∈ T and *γ*. In order to write out the problem explicitly, let us introduce the following kernel between two pairs (*x*_1_, *x*_2_) and (*x*_3_, *x*_4_):

$$\begin{array}{ccc} K_{MLPK}((x_{1},x_{2}),(x_{3},x_{4})) & = & {\langle D_{x_{1}x_{2}},D_{x_{3}x_{4}}\rangle }_{Fro}\\ & = & Trace\left((x_{1}-x_{2}){\left(x_{1}-x_{2}\right)}^{⊤}(x_{3}-x_{4}){\left(x_{3}-x_{4}\right)}^{⊤}\right)\\ & = & {\left({\left(x_{1}-x_{2}\right)}^{⊤}(x_{3}-x_{4})\right)}^{2}\\ & = & {\left(x_{1}^{⊤}x_{3}-x_{1}^{⊤}x_{4}-x_{2}^{⊤}x_{3}+x_{2}^{⊤}x_{4}\right)}^{2}. \end{array}$$

This kernel is positive definite because it is the Frobenius inner product between the matrices *D*_*ab *_representing the pairs. Moreover, although *K*_*MLPK *_is formally defined for ordered pairs only, we observe that it is invariant by permutation of the elements of each pair (e.g., when *x*_1 _and *x*_2 _are flipped). It can therefore be considered as a positive definite kernel over the set of *unordered pairs*, seen as the quotient space of the set of ordered proteins with respect to the equivalence relation of permutation among each pair. We call this kernel for unordered pairs the *metric learning pairwise kernel *(MLPK), hence the notation *K*_*MLPK*_.

In order to express the problem (8–9) in terms of the *α *variables provided by Theorem 1, we need to express the constraint *M *≽ 0 in terms of *α*. Denoting pairs of indices *t *= (*i*, *j*), Theorem 1 ensures that *M *can be written as $M=\sum_{t\in T}\alpha_{t}u_{t}u_{t}^{⊤}$. As we showed in the proof of Theorem 1, this implies that *M *is null on the space orthogonal to the linear span of (*u*_*t*_, *t *∈ T). Therefore, *M *≽ 0 if and only if *v*^⊤^*Mv *≥ 0 for any *v *in the linear span of (*u*_*t*_, *t *∈ T). This is equivalent to the fact that the |T| × |T| matrix *F *defined by $F_{t,t^{\prime }}=u_{t}^{⊤}Mu_{t^{\prime }}$ is positive semidefinite. Finally, if we denote by *F*_*t *_the |T| × |T| matrix whose (*t*_1_, *t*_2_) entry is $u_{t_{1}}^{⊤}D_{t}u_{t_{2}}=u_{t_{1}}^{⊤}u_{t}u_{t}^{⊤}u_{t_{2}}$, this is equivalent to $\sum_{t\in T}\alpha_{t}F_{t}≽0$.

Plugging the representation of Theorem 1 into (8–9), and replacing the Frobenius inner product by the MLPK kernel, we show that the problem is equivalent to

$$\underset{\alpha ,\gamma ,\zeta }{\min}\sum_{(i,j)\in T}\sum_{(k,l)\in T}\alpha_{ij}\alpha_{kl}K_{MLPK}((x_{i},x_{j}),(x_{k},x_{l}))+C\sum_{(i,j)\in T}\zeta_{ij},$$

under the constraints:

$$\begin{array}{c} \begin{array}{cc} \zeta_{ij}\geq 0, & (i,j)\in T, \end{array}\\ \begin{array}{cc} \sum_{(k,l)\in T}\alpha_{kl}K_{MLPK}((x_{i},x_{j}),(x_{k},x_{l}))\leq \gamma -1+\zeta_{ij}, & (i,j)\in ℐ, \end{array}\\ \begin{array}{cc} \sum_{(k,l)\in T}\alpha_{kl}K_{MLPK}((x_{i},x_{j}),(x_{k},x_{l}))\geq \gamma +1-\zeta_{ij}, & (i,j)\in N, \end{array}\\ \sum_{(k,l)\in T}\alpha_{kl}F_{kl}≽0 \end{array}$$

### Kernelization

An important property of the problem (13) is that the data only appear through the kernel *K*_*MLPK *_and the matrices *F*_*ij*_. Furthermore, the MLPK kernel itself (5) computed between two pairs of vectors only involves inner products between the vectors; similarly the (*t*_1_, *t*_2_)-th entry of the matrix *F*_*t *_is a product of inner products, which can easily be computed from the inner products of the data themselves. As a result, we can apply the kernel trick to extend the problem (12–13) to any data space endowed with a positive definite kernel *K*_*g*_. The resulting MLPK kernel between pairs becomes

*K*_*MLPK*_((*x*_1_, *x*_2_), (*x*_3_, *x*_4_)) = (*K*_*g*_(*x*_1_, *x*_3_) - *K*_*g*_(*x*_1_, *x*_4_) - *K*_*g*_(*x*_2_, *x*_3_) + *K*_*g*_(*x*_2_, *x*_4_))^2^,

and for any three pairs *t *= (*i*, *j*), *t*_1 _= (*i*_1_, *j*_1_), *t*_2 _= (*i*_2_, *j*_2_) in T the entry (*t*_1_, *t*_2_) of *F*_*t *_is

$$[K_{g}(x_{i_{1}},x_{i})-K_{g}(x_{i_{1}},x_{j})-K_{g}(x_{j_{1}},x_{i})+K_{g}(x_{j_{1}},x_{j})]\times [K_{g}(x_{i_{2}},x_{i})-K_{g}(x_{i_{2}},x_{j})-K_{g}(x_{j_{2}},x_{i})+K_{g}(x_{j_{2}},x_{j})].$$

### Relaxation

The problem (12–13) is a convex problem over the cone of positive semidefinite matrices that can in theory be solved by algorithms such as interior-point methods [30]. The dimension of this problem, however, is 2|T| + 1. This is typically of the order of several thousands for small biological networks with a few hundreds or thousands vertices, which poses serious convergence issues for general-purpose optimization software.

If we relax the condition *M *≽ 0 in the original problem, then it becomes the quadratic program of the SVM, for which dedicated optimization algorithms have been developed: current implementations of SVM easily handle several tens of thousands of dimensions [27]. The obvious drawback of this relaxation is that if the matrix *M *is not positive semidefinite, then it does not define a metric. Although this can be a serious problem for classical applications of distance metric learning such as clustering [11], we note that in our case the goal of metric learning is just to provide a decision function *f*(*x*, *x'*) = *d*_*M*_(*x*, *x'*) for predicting connected pairs, and negativity of this decision function is not a problem in itself. Therefore, we propose to relax the constraint *M *≽ 0, or equivalently $\sum_{(kl)\in T}\alpha_{kl}F_{k,l}≽0$ in (13), and to solve the initial problem using an SVM over pairs with the MLPK kernel (5).

## Competing interests

The authors declare that they have no competing interests.

## Authors' contributions

JPV proposed and implemented the method, carried out the experiments and drafted the manuscript. JQ and WSN helped prepare the data, participated in the design of the study and contributed to the redaction. All authors read and approved the final manuscript.
